# Inflammation-associated drug resistance and tumor growth in TNBC

**DOI:** 10.3389/fimmu.2025.1623137

**Published:** 2025-08-26

**Authors:** Arij Fouzat Hassan, Hadeel Kheraldine, Lama Abujamous, Hamda Al-Thawadi, Abdelbary Elhissi

**Affiliations:** ^1^ College of Pharmacy, Department of Pharmaceutical Sciences, QU Health, Qatar University, Doha, Qatar; ^2^ College of Medicine, Department of Basic Medical Science, QU Health, Qatar University, Doha, Qatar

**Keywords:** triple-negative breast cancer (TNBC), tumor microenvironment (TME), inflammation, cytokines, drug resistance

## Abstract

Triple-negative breast cancer (TNBC) is an aggressive and clinically challenging subtype of breast cancer characterized by the absence of hormone receptors and HER2 amplification. This molecular profile limits the effectiveness of targeted therapies, leaving chemotherapy as the mainstay of treatment a strategy often met with limited success due to rapid disease progression and high recurrence rates. Increasing evidence underscores the pivotal role of the tumor microenvironment (TME) in driving TNBC pathogenesis, particularly through chronic inflammation and cytokine dysregulation. Inflammatory cytokines such as TNF-α, TGF-β, IL-6, and IL-10 orchestrate a complex network of cellular interactions that remodel the TME into an immunosuppressive niche. This inflammatory landscape not only promotes tumor cell proliferation and metastasis but also compromises antitumor immune responses and contributes to therapeutic resistance. Recent preclinical and clinical studies have explored the therapeutic potential of targeting cytokine signaling to disrupt this inflammatory axis and overcome resistance. In this review, we critically examine the multifaceted interplay between cytokines, inflammation, and the TME in TNBC, with a focus on mechanisms of resistance. We further evaluate current and emerging therapeutic approaches targeting the inflammatory axis, highlighting both the promise and the complexities of this evolving landscape.

## 1 Introduction

**Figure 1:**
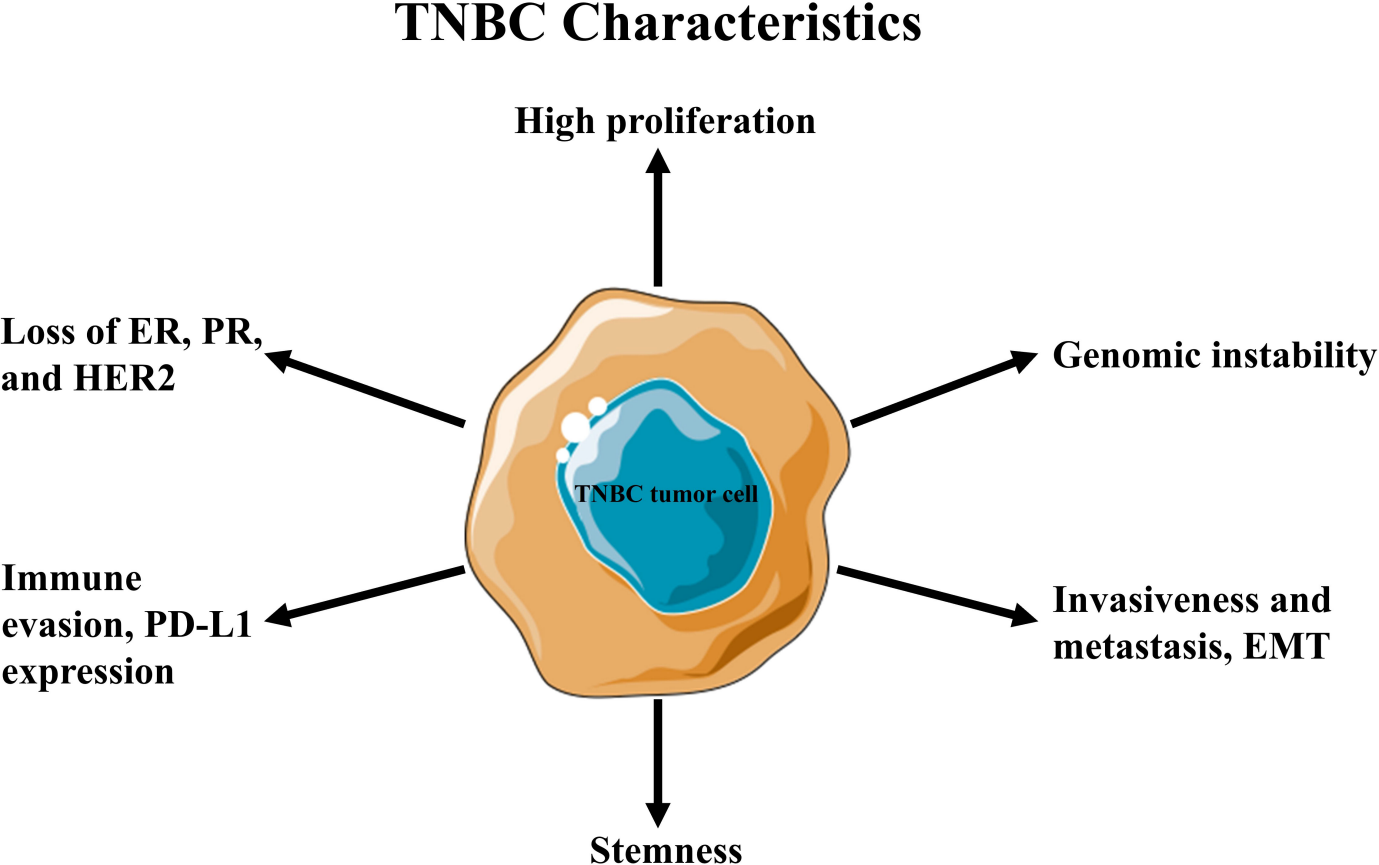
Hallmark characteristics of triple-negative breast cancer (TNBC).

Triple-negative breast cancer (TNBC) is an aggressive subtype lacking the expression of estrogen receptor (ER), progesterone receptor (PR), and epidermal growth factor receptor type 2 (HER2) (1, 2). It accounts for approximately 15–20% of all breast cancer cases globally (~170,000 annually) (3). It disproportionately affects premenopausal women, with higher prevalence among African American women and those carrying pathogenic breast cancer susceptibility genes, such as BRCA mutations (4).TNBC is characterized by rapid tumor growth, early metastasis (often to the lungs, liver, and brain), and a higher likelihood of recurrence, particularly within the first three to five years after treatment, where the survival rate is less than 10% (1, 2). Its clinical behavior varies due to underlying molecular heterogeneity, influencing therapeutic response and outcomes at both early and advanced stages (5). The absence of targetable receptors makes TNBC unresponsive to hormonal therapies such as tamoxifen and aromatase inhibitors, as well as HER2-targeted treatments like trastuzumab (**Figure 1**). As a result, chemotherapy remains the primary systemic treatment option, highlighting the need for a deeper understanding of TNBC’s unique biology and tumor microenvironment (TME) (6, 7).

A key contributor to TNBC progression and resistance is the TME a complex and dynamic network of stromal cells, immune cells, extracellular matrix, and signaling molecules (8). Cytokines within the TME drive inflammation, immune evasion, angiogenesis, and epithelial-mesenchymal transition (EMT) in TNBC, supporting tumor survival and metastasis and making them attractive candidates for therapeutic intervention (9, 10). Moreover, immunosuppressive components such as tumor-associated macrophages (TAMs), myeloid-derived suppressor cells (MDSCs), and regulatory T cells (Tregs) exacerbate resistance by shielding tumor cells from immune attack (11, 12).

Recent studies have highlighted the therapeutic potential of targeting cytokine signaling and the inflammatory TME to restore treatment sensitivity in TNBC. Strategies such as cytokine inhibitors, immune checkpoint blockade, and agents that reprogram the TME have shown promise in preclinical and clinical settings, offering hope for more effective and durable treatments (13, 14).

This review explores the interplay between inflammatory cytokines, the TME, and drug resistance in TNBC, with a focus on mechanisms that drive progression and immune suppression. We also evaluate emerging interventions aimed at disrupting this axis to enhance therapeutic efficacy and overcome resistance.

### 1.1 Tumor microenvironment in TNBC

TME refers to the complex and dynamic milieu of cells, extracellular matrix, and signaling molecules that surround and interact with tumor cells (15). Since TME is necessary for the growth and invasiveness of cancer cells, it has emerged as a promising target for the development of novel cancer therapeutics (16). The TME is comprised of a variety of cell types, including cancer cells, immune cells, endothelial cells, pericytes, and cancer-associated fibroblasts (CAFs) (17). These cells can both support and impede the growth and spread of tumors (18). The endothelial cells, as well as CAFs, secrete cytokines to regulate EMT (19). EMT-triggered tumor cells produce immunosuppressive cytokines, leading to an immunosuppressive TME (19).

To maintain their own survival and growth, tumor cells regularly actively change their microenvironment, resulting in a dynamic and complicated interplay between neoplastic cells and the TME (20). TME can be divided into immunosuppressive and immunoreactive categories based on its contribution to the immune response (21). The predominant immune cells within the tumor microenvironment are lymphocytes, commonly referred to as tumor-infiltrating lymphocytes (TILs), which are frequently observed in solid tumors. TILs consist of various subtypes, with CD3^+^ T cells being the most abundant in solid tumors. While CD20^+^ B cells may also be present, their infiltration is relatively uncommon. Indeed, these various TIL subtypes play diverse roles in the management of breast cancer and contribute to immunomodulation through distinct pathways (22).

In the early stages of tumor development, immune cells can recognize and eliminate highly immunogenic cancer cells (23). However, under conditions such as cellular stress, infection, or inflammation, cancer cells can evade immune surveillance, allowing tumor progression to occur unchecked (24, 25). Increasing evidence has highlighted inflammation as a key factor in shaping the TME and promoting cancer development.

### 1.2 Inflammation in TME of TNBC

Inflammation significantly contributes to the emergence and development of TNBC, particularly when chronic inflammation permeates the TME. Chronic inflammation can affect different elements of TNBC etiology and is characterized by a protracted and dysregulated immune response. Recent studies strongly imply that chronic inflammation is associated with aberrant metabolism, which is most likely to alter the TME and promote cancer-associated signaling cascades (26–28). The immune system mounts a response to harmful agents, such as pathogens, irradiation, or tissue injury, resulting in inflammation (29).

In many instances, the development and spread of cancer are correlated with inflammation. Since cells responsible for inflammation-induced cancers are often stable and resistant to current anticancer therapies, targeting inflammation has emerged as a promising therapeutic strategy. Inflammation that arises within tumors is associated with several risk factors, including pathogenic infections, autoimmune diseases, lifestyle factors, smoking, and excessive alcohol consumption. Additionally, cancer-intrinsic or cancer-induced inflammation can originate from cancer-initiating mutations, which recruit and activate inflammatory cells, ultimately supporting tumor growth (30).

The primary goal of the inflammatory response is to eliminate the foreign substance that interferes with tissue homeostasis. During acute inflammation, cellular and molecular interactions restore the homeostatic condition in the typical physiological environment. On the other hand, when inflammation is not resolved adequately, it leads to chronic inflammation (31). Chronic inflammation can increase the risk of cancer by supplying bioactive molecules from invasive cells into TME, such as cytokines, to prevent proangiogenic factors, and ECM-modifying enzymes like metalloproteinases that promote EMT and facilitate other carcinogenesis programs (32).

In TNBC, pro-inflammatory pathways, tissue damage, infection, and other factors can contribute to chronic inflammation. Cytokines are pivotal mediators in chronic inflammation, orchestrating the communication between immune cells and influencing cellular behaviors in TME (33). TNBC has been associated with many cytokines, including those necessary for tumor growth, such as TGF-β, TNF-α, IL-6, and IL-10 (34, 35). Understanding the mechanisms behind the interactions between TNBC cells and their microenvironment is therefore essential for the development of effective cancer therapies.

For the purpose of developing effective therapeutic strategies that specifically target these pathways to improve outcomes for TNBC patients, it is crucial to understand the role of cytokines in TNBC-associated immunosuppression and drug resistance. This review explores the mechanisms by which cytokines act within the TME, highlighting the inflammatory responses that contribute not only to immune evasion but also to the promotion of therapeutic resistance in TNBC.

## 2 Cytokines in TNBC

Cytokines are small secreted proteins of size less than 40kDa produced by cells involved in the immune response (36). Although different types of cells produce cytokines, helper T cells and macrophages are the chief producers. Schwann cells, mast cells, endothelial cells, and resident and recruited macrophages may produce cytokines in and via peripheral nerve tissue during normal and pathological processes (37). The release of pro-inflammatory cytokines stimulates and activates immune cells, as well as induces the release of further cytokines (36).

**Figure 2:**
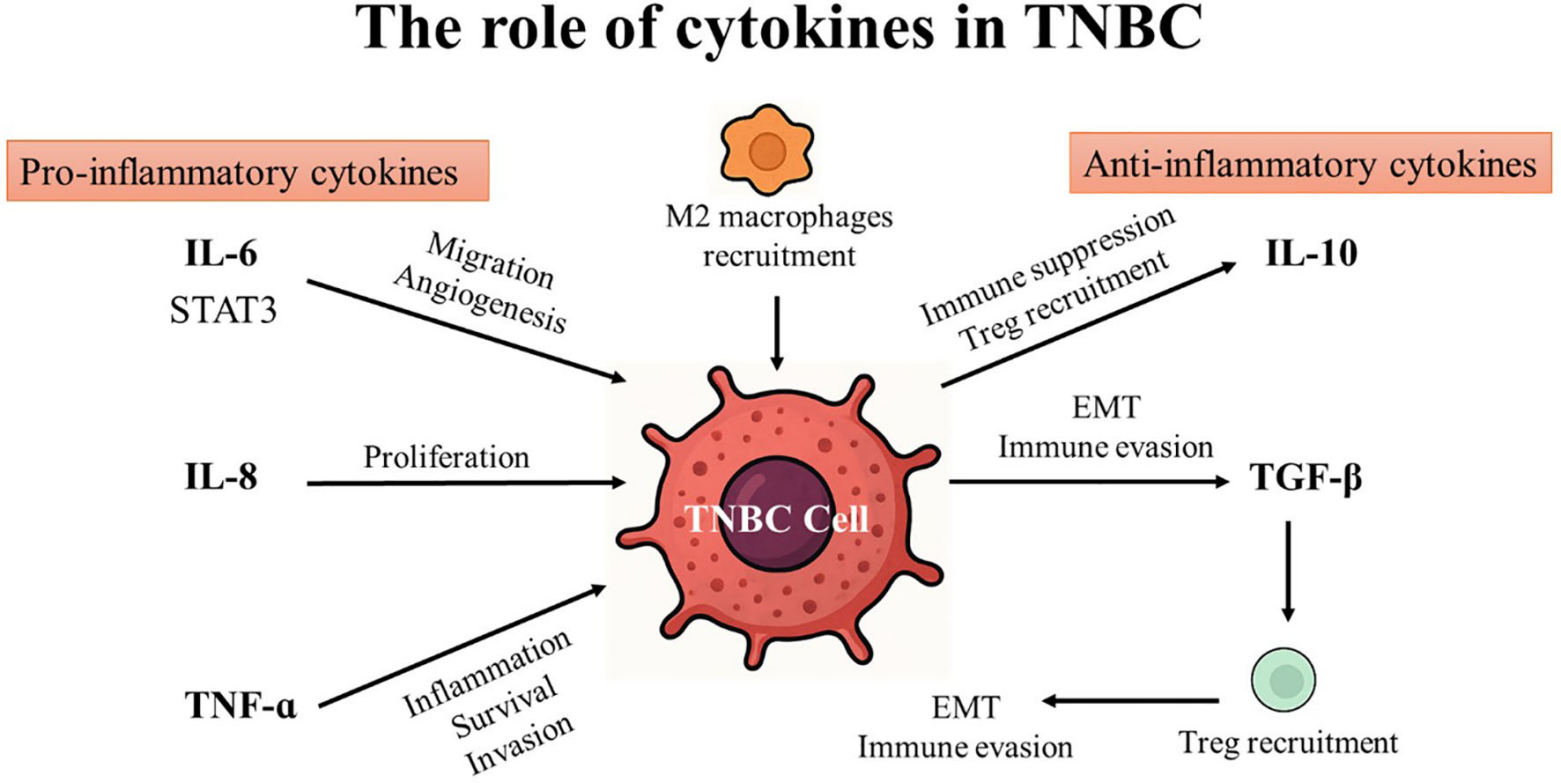
The role of pro- and anti-inflammatory cytokines in triple-negative breast cancer (TNBC).

Cytokines are generated in response to a wide variety of cellular stressors, such as inflammation, infection, and damage brought on by carcinogens. These conditions trigger cytokines to activate a host response targeted at reducing cellular damage and managing cellular stress. Effective damage containment encourages tissue repair, but failure to treat the damage might result in cytokine production that persists and worsens tissue loss. As a result, host responses to cellular stress may influence various phases of cancer development and progression (38). Cytokines enable paracrine and autocrine immune system cell communication over short distances in the innate and adaptive immune systems. Since the immune system is capable of identifying and eliminating cancer cells, research has focused on using cytokines to treat cancer during the past few decades (39). Cytokines play an important role in cancer by regulating cell differentiation and proliferation, tumor development, progression, and immune response (40). Generally, cytokines can be categorized into pro-inflammatory and anti-inflammatory based on their function and the type of immune responses they regulate (41, 42). (see **Table 1**; **Figure 2**).

**Table 1:** Key cytokines in triple-negative breast cancer (TNBC) tumor microenvironment (TME).

Cytokine	Source in TME	Pro-/Anti-inflammatory	Key functions in TNBC	Therapeutic targeting	Reference
TGF-β	Cancer cells, CAFs, immune cells	Dual (context-dependent)	Induces EMT, promotes metastasis, immunosuppression, ECM remodeling	TGF-β inhibitors (e.g., galunisertib)	(108–115)
TNF-ɑ	Macrophages, cancer cells	Pro-inflammatory	Promotes inflammation, NF-kB activation, EMT, and metastasis	TAK1 or NF-kB inhibitors, anti-TNF antibodies	(129–136)
IL-6	Tumor cells, stromal cells, immune cells	Pro-inflammatory	Activates STAT3, promotes survival, angiogenesis, and chemoresistance	STAT3 inhibitors, IL-6R blockade (e.g., tocilizumab)	(139–142)
IL-10	Regulatory T cells, M2 macrophages, tumor cells	Anti-inflammatory (immunosuppressive)	Suppresses antigen-presenting cells, promotes Tregs and immune evasion	IL-10 neutralization or modulation of M2 macrophages	(148–152)

For instance, Th1 cells, CD4+ cells, macrophages, and dendritic cells release pro-inflammatory cytokines including Interleukins (IL), interferon (IFN), and tumor necrosis factor (TNF). The three primary cytokines that encourage inflammation are IL-6, IL-10, and TNF. These cytokines communicate their signals through type I cytokine receptors (CCR1), which are physically distinct from other cytokine receptor types. They are crucial for modulating the immune system and for coordinating cell-mediated immune responses. Pro-inflammatory cytokines typically affect immune cell proliferation, activation, differentiation, and homing to the sites of infection to control and eradicate intracellular pathogens (99).

### 2.1 Pro-inflammatory cytokines in TNBC

Pro-inflammatory cytokines have a significant impact on the development of TME in TNBC. Numerous stromal cells and immunological cells produce these cytokines within the TME, and their presence contributes to chronic inflammation within the tumor milieu. Pro-inflammatory cytokines act as key signaling molecules, orchestrating complex interactions between malignant and immune cells, and stromal components. Through their actions, they can modulate tumor cell survival, proliferation, angiogenesis, and immune evasion mechanisms (100, 101). Pro-inflammatory cytokines also affect immune cell recruitment, activation, and performance inside the TME, affecting the anti-tumor immune response. Their ability to shape the TME creates a favorable environment for tumor growth, invasion, and metastasis. It is crucial to comprehend how pro-inflammatory cytokines interact with the TME to create targeted therapeutics that disrupt tumor-promoting pathways and enhance clinical outcomes (102). TGF-β, TNF-α, IL-6, and IL-10 are a few pro-inflammatory cytokines that regulate TME in TNBC and promote cancer progression and invasiveness (103).

### 2.2 Anti-inflammatory cytokines in TNBC

**Figure 3:**
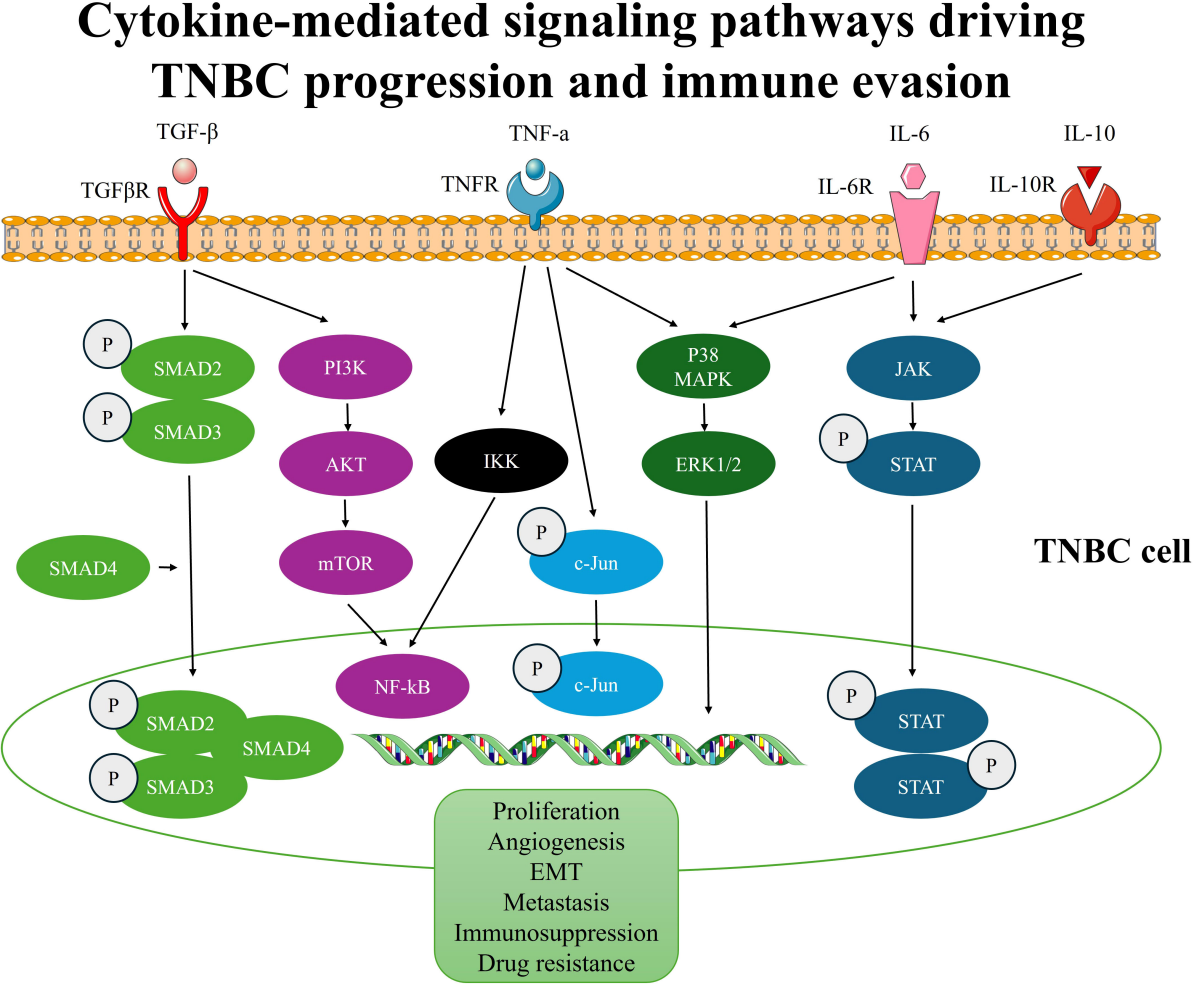
Cytokine-mediated signaling pathways driving TNBC progression and immune evasion.

Anti-inflammatory cytokines play a critical role in maintaining immune homeostasis and regulating inflammation. Key cytokines in this group include interleukin-10 (IL-10), transforming growth factor-beta (TGF-β), and interleukin-35 (IL-35) (104). These cytokines suppress excessive immune activation, protect tissues from damage during inflammation, and promote wound healing (105). However, in the context of cancer, their immunosuppressive properties can paradoxically facilitate tumor progression. For example, TGF-β suppresses cytotoxic T cell and natural killer (NK) cell activity while promoting regulatory T cell (Treg) expansion, contributing to immune evasion by cancer cells. TGF-β is also involved in EMT, a key process in cancer metastasis (106). Further, IL-10 inhibits the activation of antigen-presenting cells and effector T cells, thereby impairing anti-tumor immune responses and creating an immunosuppressive TME (105). Similarly, IL-35, though less studied, has also been shown to enhance Treg function and inhibit anti-tumor immunity (107). Collectively, anti-inflammatory cytokines contribute to the establishment of a tolerogenic TME that favors tumor growth, metastasis, and resistance to therapies. Targeting these cytokines or their signaling pathways holds promise for reversing immunosuppression and enhancing anti-cancer immune responses (**Figure 3**).

### 2.3 Transforming growth factor-β

TGF-β is a versatile cytokine essential for many physiological and pathological processes. It is a member of the TGF-β superfamily of cytokines and includes various isoforms such as TGF-β1, TGF-β2, and TGF-β3. It is a powerful regulator of cell development, differentiation, apoptosis, immune responses, tissue development, and homeostasis. TGF-β is produced by many cell types, including immune cells, fibroblasts, and epithelial cells. It exists as an inactive precursor that needs to be activated to have a biological impact. Upon activation, TGF-β binds to its receptors on the cell surface, initiating downstream signaling pathways that regulate gene expression and cellular responses. TGF-β has a wide range of functions based on the cell type, microenvironment, and the disease (108, 109).

TGF-β functions as a tumor suppressor under normal physiological circumstances by preventing cell growth, causing cell cycle arrest, and encouraging apoptosis. It is also involved in tissue repair and immune regulation, contributing to wound healing and immunosuppressive effects. TGF-β signaling dysregulation, on the other hand, is linked to the onset and development of several illnesses, including cancer (110). TGF-β displays a dual role; while it suppresses tumor growth in the early stages of tumorigenesis, it promotes tumor development and metastasis in advanced stages. Moreover, it has the ability to trigger EMT, an important event driving tumor cell invasion and metastasis. In addition, TGF-β also contributes to immunosuppression by inhibiting immune cell function and promoting regulatory T-cell differentiation (111, 112).

In other words, a crucial TME regulator, TGF-β, is involved in the complex interactions between stromal cells and tumors. Malignant cells and cells that exist in TME can produce TGF-β, leading to autocrine and paracrine signaling. TGF-β signaling influences multiple aspects of the TME, including immune cell infiltration, angiogenesis, extracellular matrix remodeling, and EMT. One of the critical functions of TGF-β in the TME is its immunomodulatory role. TGF-β suppresses the activity of immune cells, such as cytotoxic T cells, NK cells, and dendritic cells, impairing their anti-tumor functions. It promotes the differentiation and activity of immunosuppressive Tregs and myeloid-derived suppressor cells (MDSCs). This immunosuppressive environment allows tumors to evade immune surveillance and promotes immune tolerance (113, 114).

In TNBC, the TGF-β cascade is often dysregulated, resulting in aberrant activation and an imbalance in its effects within the TME. TGF-β signaling in TNBC exerts diverse effects on different components of the TME, influencing tumor progression and therapeutic resistance (115). In highly complex diseases like TNBC, the most important factor that controls carcinogenesis and treatment response is the signaling cascades between tumors and adjacent cell populations, such as ECM and CAFs. Despite the lack of knowledge regarding TGF-β’s role in TNBC, CAF in axillary lymph nodes causes BC metastases via comparable processes, including EMT that utilizes the TGF-β pathway. The aggressive BC phenotype is stimulated by the interplay of TGF-β pathways, specifically the downregulation of TGF-β receptors, which creates an irrational association with patient outcomes (116). According to a recent study, intratumorally high levels of TGF-β1 were observed in more than 50% of TNBC as opposed to non-TNBC, suggesting a possible function for TGF signaling in the biology of TNBC (117). Additionally, an *in-vitro* study showed that TGF-β1 was discovered to increase MDA-MB-231 cells’ propensity to form tumors by triggering the SMAD2 and P38 signaling pathways (118). Collectively, several investigations show a connection between TGF-β and TNBC’s aggressiveness, poor disease-free survival, and metastatic status (115, 119, 120). The TGF-β signaling cascade is a well-known oncogenic pathway in TNBC that promotes tumor stemness and EMT-mediated cancer plasticity. The stimulation of TGF-β signaling results in the production of EMT-activating transcription factors (EMT-ATFs), which function as molecular switches for the EMT and stemness nuclear reprogramming (121, 122). TGF-β typically acts as a tumor promoter in late-stage cancers by promoting EMT (123). In addition, the TGF-β signaling pathway is initiated when TGF-β ligands bind to type II receptors (TGFBR2) on the cell surface. This binding leads to the recruitment and activation of type I receptors (TGFBR1), resulting in phosphorylation and activation of downstream signaling molecules called SMADS (124). The TGF-β signaling pathway can be canonical or non-canonical. During the canonical TGF-β signaling, TGF-β is initiated through the activation of its receptor and the recruitment of phosphorylated SMADs (SMAD2/SMAD3), which form complexes with SMAD4 and translocate to the nucleus (124). Once within the nucleus, these complexes control the transcription of target genes involved in a variety of cellular activities, including EMT by interacting with transcription factors and co-regulators (125). However, through a non-canonical TGF-β axis, it can also control physiological and pathological responses. The non-canonical cascade, for instance, also includes the extracellular signal-regulated kinases (ERK), p38, and c-Jun N-terminal kinase (JNK), as well as the cell survival mediator’s protein kinase B (AKT), mammalian target of rapamycin (mTOR), and nuclear factor kappa-B (NF-kB) (126). The development of cancer-related disease is significantly influenced by the dysregulation of these non-canonical TGF-β signaling pathways. Further, overexpression of the c-Myc gene, along with activation of the p15, p21, and SMADs, eliminates the antitumor impact of TGF-β on cell development, resulting in the evolution of tumors (127). Furthermore, TGF-β signaling in TNBC is involved in angiogenesis and the remodeling of the ECM. Increased tumor vascularization results from TGF-β’s stimulation of the synthesis of pro-angiogenic substances, including vascular endothelial growth factor (VEGF), and enhancement of endothelial cell migration and tube formation. Moreover, TGF-β stimulates the recruitment of ECM proteins, including fibronectin and collagen, and modulates the activity of matrix metalloproteinases (MMPs), which contribute to the breakdown of the ECM and the infiltration of malignant cells (128).

### 2.4 Tumor necrosis factor

TNF-α is a cytokine that is primarily generated by immune cells like macrophages upon activation. TNF-α participates in different physiological and pathological processes, and its dysregulation is implicated in several diseases. TNF-α is a cytokine that promotes inflammation and is essential for the initiation and progression of the inflammatory response (129). TNF-α stimulates the recruitment of other inflammatory molecules, such as interleukins and chemokines, and promotes the migration of immune cells to the site of inflammation. TNF-α production can cause chronic inflammation and tissue damage, even though it is necessary for immune responses and host defense. Abnormal TNF-α signaling has been associated with various autoimmune diseases. TNF-α encourages the activation of immune cells, the release of inflammatory mediators, and the death of healthy tissues under abnormal circumstances (129).

In cancer, TNF-α has an essential role, exhibiting both pro-tumor and anti-tumor effects depending on the context and stage of cancer development (130). It can induce apoptotic proteins in certain malignant cells, particularly in the early stages of tumor development. It can also promote inflammation and immune responses that can aid in the elimination of cancer cells. Nevertheless, TNF-α can exert pro-tumor effects by promoting tumor cell proliferation, survival, and angiogenesis. It can increase the production of cytokines and growth factors that encourage the development and invasion of tumor cells (130). TNF-α may also stimulate the growth of a microenvironment that is supportive of tumors by luring immune cells that inhibit anti-tumor immune responses and fasten tumor development. The fact that TNF-α may affect TME further complicates the dual role that it plays in cancer. TNF-α may affect the recruitment and activation of immune cells within the tumor, resulting in a dynamic interaction between immune responses that promote and suppress tumor growth. The relative importance of these conflicting effects can define TNF-α ‘s overall influence on tumorigenesis (131).

In BC, patients’ biopsies revealed high TNFα-mRNA and protein expression, particularly in those with a worse prognosis (132). Recently, the pro-metastatic function of TNF-α and its involvement in the EMT for tumor cell migration to initiate metastasis were reported (133). In fact, it is considered one of the inflammatory cytokines produced by TME and is linked to metastasis and aggressiveness in several malignancies, including TNBC (134). The role of TNF-α in survival and proliferation has produced contradictory outcomes. Depending on the cell type and cellular milieu in which it is detected, TNF-α can cause apoptosis and hasten the formation of tumors. TNF-α, on the other hand, can stimulate c-Jun, which in turn activates pro-apoptotic pathways and downregulates anti-apoptotic genes, resulting in apoptosis. However, the same study showed that TNF-α may encourage tumor growth (135, 136). During tumorigenesis, and through the activation of the NF-kB and p38/MAPK pathways, TNF-α activates the signal transducer and activator of transcription 3 (STAT3), a well-known transcription factor classified as an oncogene. This cascade creates a positive feedback loop, as when STAT3 is activated, HBXIP (Hepatitis B Virus X-Interacting Protein) is expressed more abundantly, which increases the level of TNFR1. As a result, TNFR1 binds to TNF-α, maintaining the activation of the p38/MAPK and NF-kB pathways. Additionally, the membrane glycoprotein MUC4 can promote cell proliferation in TNBC cell lines by upregulating cyclin D1 expression and mediating β-catenin (137). Another *in-vitro* study showed that TNF-α induces cancer metastasis by the generation of MMP, which can change the ECM during metastasis. It is important to highlight that the TGF-β-activated kinase 1 (TAK1) complex is essential for TNF-α-mediated MMP9 synthesis in this procedure (138).

### 2.5 Interleukin-6

IL-6 is a glycosylated protein also known as B cell differentiation factor (BSF-2), which stimulates B-cell maturation into antibody-producing cells. It has important functions in maintaining hematopoietic progenitor cells, the cardiovascular and nervous systems (139). Additionally, IL-6 contributes to both acute and chronic inflammation. At the location of tissue injury, IL-6 is released during an immunological response and aids in directing immune defense. It increases the liver’s ability to produce acute-phase proteins, including C-reactive protein (CRP), which aids in the identification and removal of infections. Additionally, IL-6 encourages immune cell activation and differentiation, including B and T cell differentiation, and modifies the balance between pro-inflammatory and anti-inflammatory responses (140).

In the TME of cancer, IL-6 is important. It is produced by a variety of cell types in TME, including stromal cells, immune cells, and cancer cells themselves. The growth, development, and immunological responses of tumors can be significantly impacted by the presence of IL-6 in TME (141). Essentially, in TME, the signaling pathway of IL-6 is considered a malevolent player, due to its function in cancer development and progression. Based on animal studies, chronic IL-6 signaling is highly associated with carcinogenesis. Through a variety of downstream mediators, IL-6 exerts an intrinsic effect on tumor cells that promotes cancer cell survival, proliferation, and metastasis. Furthermore, IL-6 can exert extrinsic effects on other cells in the complex TME to maintain a pro-tumor milieu by increasing angiogenesis and tumor immunity evasion (142).

IL-6 was discovered to be overexpressed in BC patients’ blood and tumor sites, which is often associated with poor prognosis and decreased survival. IL-6 can influence every stage of the BC process by regulating proliferation, apoptosis, metabolism, survival, angiogenesis, and metastasis (143). In the context of TNBC, IL-6 is expressed in 50% of TNBC cases (144). IL-6, one of the main mediators of the inflammatory response, has a substantial impact on both the defense mechanisms of the host immune system and the control of cellular development. The transmission of IL-6 signals triggers STAT3 and involves both the cell-surface IL-6 receptor (IL-6R) and the soluble IL-6 receptor (sIL-6R). In TNBC patients, STAT3 activation is associated with lower survival rates and chemoresistance (145). According to a new study, the STAT3 pathway, which is controlled by IL-6 under genotoxic stress, is stimulated by the stimulator of interferon genes (STING), which circumvents immune suppression by raising PD-L1 in TNBC. The activation of STING pathways in tumor cells and immune cells recruited in the TME is of significant interest since it may help to alter tumor intrinsic cell survival and death pathways. STING induces the production of IL-6 in TNBC cells under genotoxic stress, which binds to its specific receptor and promotes phosphorylation of the tyrosine (705) residue of STAT3. This suggests that the IL-6/STAT3 pathway is triggered via the STING pathway (146). Another study illustrated that TNBC cells activate the IL-6-STAT3 signaling pathway, which increases levels of the chemokines, VEGF, and chemokine (C-C motif) ligand 5 (CCL5) and encourages lymph node angiogenesis and lung extravasation of tumor cells (147).

### 2.6 Interleukin-10

Interleukin-10 (IL-10) is an immunoregulatory cytokine that plays a critical role in modulating immune responses and maintaining immune homeostasis. IL-10 acts on multiple cells types and exerts both anti-inflammatory and immunosuppressive effects. One of the key functions of IL-10 is its ability to dampen immune responses and limit inflammation. TNF-α and IL-6 are two examples of pro-inflammatory cytokines whose synthesis and activity are inhibited by IL-10. Additionally, it prevents immune cells like T cells, NK cells, and antigen-presenting cells from activating and functioning. By reducing the inflammatory response, IL-10 helps prevent tissue damage caused by excessive or prolonged immune activation. IL-10 is particularly important in regulating immune responses at mucosal surfaces, such as the gastrointestinal tract. It helps maintain immune tolerance to commensal microorganisms and food antigens while still allowing for effective immune responses against pathogens. Dysregulation of IL-10 signaling can contribute to chronic inflammation and diseases, including cancer (148, 149). It is well recognized that IL-10 has dual roles as anti-tumor and pro-tumor. Tumor regression activity is demonstrated by IL-10. The anti-tumor effect of IL-10 depends on CD8+ or CD4+ T cell activity, contrary to some who have suggested that the anti-tumor action of IL-10 is caused by increased NK cell activity (150). The tumor can thwart the host immune system’s ability to eliminate it by using IL-10 production at the tumor location. It is essential for local cancer immunotherapy that IL-10 has the capacity to stop an immune response from growing at the tumor site (151). In fact, cancer cell proliferation and metastasis are enhanced by IL-10 through the regulation of antitumor immunity (152).

Although little is known about how IL-10 impacts TNBC, there is mounting evidence that it plays a role in the growth of cancer. However, it was shown that TNBC samples had a significant expression of IL-10 (153). IL-10 exerts both direct and indirect effects on TNBC cells and TME. One mechanism by which IL-10 may influence TNBC is through IgG4. It was reported that IgG4+B cells are linked to higher tumor recurrence and unfavorable patient prognosis in TME of TNBC. IgG4+B cell presence correlates with IL-10 tumor expression. These findings imply that, due to the presence of IL-10 overexpression, TNBC may offer a TME that supports class change to the IgG4 subtype. Furthermore, hormone-receptor negative tumors, higher-grade tumors, and locally advanced cancer are more prevalent in TNBC patients with IL-10 expression in their malignancies (154). Another mechanism responsible for IL-10 overexpression is through the production of proinflammatory cytokines, M1 and M2-type macrophages. M1-macrophages are linked to an inflammatory response and trigger a Th1 immune response. M2-type macrophages, which are linked to tumor progression, secrete IL-10 and suppress the Th1 immune response, further inducing tumor invasion and metastasis. Additionally, angiogenic factors like VEGF can be secreted by M2-type macrophages to support tumor angiogenesis and supply nutrients and channels for metastasis. Additionally, TNBC cells also release IL-10 and M-CSF to promote the polarization of M1-type macrophages into M2-type states (155).

Collectively, these findings suggest that cytokines in the TME of TNBC function within a tightly regulated yet dynamic signaling network that promotes survival, plasticity, and immune evasion (156). We propose a mechanistic model in which chronic cytokine signaling—particularly via IL-6, TGF-β, and TNF-α—drives a feedforward loop that reinforces tumor aggressiveness. Through activation of key pathways such as STAT3, NF-κB, and SMAD, these cytokines promote EMT, reprogram immune cell behavior (e.g., M2 macrophage polarization), and sustain transcriptional plasticity in tumor cells (157–159). Notably, TGF-β may initiate early transcriptional reprogramming, while IL-6–mediated STAT3 activation supports long-term drug resistance by maintaining survival and angiogenic signaling (43). These feedback circuits, often enhanced by stress responses such as STING or hypoxia-induced HIF-1α, create a self-sustaining cytokine–TME axis that contributes to therapeutic failure (44, 45). Understanding these interconnected signaling events may offer new avenues for combination therapies targeting multiple cytokine-driven nodes simultaneously.

### 2.7 Metastatic potential of TNBC and cytokines involvement

TNBC is notably aggressive, with a high propensity to metastasize to the bone, lungs, liver, and brain (46, 47). This metastatic potential largely determines patient prognosis and therapeutic failure (48). Metastasis is a complex, multi-step process that includes local invasion, intravasation, survival in circulation, extravasation, and colonization at distant sites. Each step is governed by dynamic interactions between TNBC cells, the TME, and host immune responses (13, 49).

Early in metastasis, hypoxia, acidosis, and mechanical stress in the primary tumor promote the secretion of cytokines and recruitment of bone marrow-derived immune cells (50, 51). These events facilitate extracellular matrix (ECM) remodeling, angiogenesis, and EMT. Key cytokines such as TGF-β, IL-6, and TNF-α drive EMT by activating SMAD, STAT3, and NF-κB pathways, loosening cell–cell adhesion and promoting a migratory phenotype (52–54). Additionally, cytokines such as CXCL12, VEGF, and MMP-9 contribute to the formation of pre-metastatic niches, conditioning distant organs and recruiting supportive stromal and immune cells (55, 56).

Organ-specific metastasis (organotropism) in TNBC involves both tumor-intrinsic traits and microenvironmental cues (57). For instance, bone metastasis involves integrins (αvβ3, α5β1), TGF-β, HIF-1α, and MMP-mediated signaling (58, 59). Brain colonization requires breaching the blood-brain barrier via CXCR4, VEGF, and COX-2, aided by astrocyte-derived cytokines and WNT pathway activation (60, 61). Lung and liver metastases are facilitated by metabolic reprogramming and cytokine-driven ECM modulation, including pyruvate carboxylase, LOX, and β-catenin-independent WNT signaling (62, 63).

Emerging regulators such as non-coding RNAs (miRNAs and lncRNAs) and ferroptosis-related genes are also implicated in metastasis (64). Notably, miRNAs like miR-125b suppress EMT and metastatic behavior, while ferroptosis, an iron-dependent form of cell death, interacts with cytokine signaling and redox balance, representing a novel axis of metastatic regulation in TNBC (65, 66).

## 3 TME targeted therapeutics in TNBC

Compared to other BC subtypes, TNBC typically manifests as a high-grade invasive ductal carcinoma with a higher rate of early recurrence, frequent distant metastases, and is associated with worse prognosis. Although there is progress in understanding tumor biology, clinical outcomes for TNBC are, regrettably, still not sufficient. This fact underscores how urgently better therapeutic interventions must be developed for TNBC patients (67). Further, the lack of targetable receptors renders TNBC unresponsive to hormonal therapies such as tamoxifen and aromatase inhibitors, as well as HER2-targeted therapies like trastuzumab. This limitation leaves chemotherapy as the main systemic treatment option and underscores the need for a deeper understanding of its distinct biology and TME (**Table 2**).

**Table 2:** Summary of therapeutic strategies targeting the tumor microenvironment (TME) in triple-negative breast cancer (TNBC).

Approach	Mechanism	Examples	Key Trials/Findings	Challenges	References
Immune Checkpoint Inhibitors (ICIs)	Block PD-1/PD-L1 or CTLA-4 to restore T cell activation and immune response; effective in PD-L1+ TNBC	Pembrolizumab, Atezolizumab, Durvalumab + Tremelimumab	KEYNOTE-012, IMpassion130, JAVELIN, TBCRC 043	Low response in unselected patients, resistance, and need for biomarkers	(13, 43–55)
TAMs-Targeting Agents	Reprogram M2 macrophages to M1 or deplete TAMs; reduce immune suppression	PLX3397, Eganelisib, CCR2/CCR5 inhibitors, CD47 blockers, Imiquimod, Zoledronic acid	MARIO-3 trial, CD47+PD-L1 bispecific antibodies	Limited efficacy as monotherapy, better in combination; patient stratification needed	(56–67)
Anti-Angiogenic Agents	Inhibit VEGF and normalize vasculature to improve drug delivery and immune infiltration	Bevacizumab, Sorafenib, Aflibercept, Ramucirumab	VEGF blockade induces hypoxia, activating HIF-1Î± and alternative pro-tumor pathways	Hypoxia leads to resistance; cytokine-mediated immune suppression	(8, 68–95)
Metabolic & Other Emerging Therapies	Target cholesterol biosynthesis, autophagy, TGF-Î², enhance TILs, and use ADCs or CAR-T cells	LYN-1604, S63845, Galunisertib, CAR-T, Sacituzumab govitecan (ADC)	Sacituzumab approved for relapsed TNBC, clinical trials ongoing for others	Need for better delivery, combination order/timing, and new targets	(44, 96–98)

### 3.1 Immune checkpoint inhibitors in TNBC

Several targeted therapeutic approaches have shown promise in addressing the TME of TNBC, such as immune checkpoint inhibitors (ICIs), which aim to unleash the anti-tumor immune response and transform the TME from an “immune cold” to an “immune hot” state (8). Ongoing research and clinical trials continue to expand the landscape of targeted therapeutics, offering hope for improved outcomes in TNBC patients (68, 69). The two most widely studied ICIs function by blocking the PD-1/PD-L1 axis or CTLA-4. PD-L1 was shown to be present in 20-30% of TNBC cases, which made PD-1/PD-L1 inhibitors effective against TNBC. ICIs such as pembrolizumab and atezolizumab block the interaction between PD-1 and its ligand, restoring T cell activation and promoting an effective immune response (70).

Clinical trials, including the KEYNOTE-012 study, demonstrated that pembrolizumab showed manageable safety and promising antitumor activity in patients with PD-L1-positive advanced TNBC (71). Further, the phase I trial of JS001, an anti-PD-1 monoclonal antibody, showed encouraging safety and preliminary efficacy in patients with advanced TNBC who had undergone multiple lines of systemic therapy, suggesting its potential as a treatment option for this heavily pretreated population (72). Further, the JAVELIN study evaluated the outcome of avelumab, an anti-PD-L1 monoclonal antibody, in patients with metastatic TNBC. It was found that while avelumab demonstrated manageable safety, its overall efficacy as monotherapy was limited (73). These clinical trials highlighted the need for combination approaches or better patient selection strategies, particularly in PD-L1-positive.

CTLA-4 inhibitors, though less explored in TNBC, have shown promise in preclinical studies and early-phase trials. By targeting CTLA-4, these ICIs enhance T cell priming and activation, boosting antitumor immune responses. Dual blockade of PD-1/PD-L1 and CTLA-4 pathways is being investigated as a strategy to achieve synergistic effects, particularly in TNBC cases with cold or poorly immunogenic tumors (74). For instance, a pilot study of durvalumab (anti-PD-L1) and tremelimumab (anti-CTLA-4) in metastatic TNBC demonstrated manageable safety and provided insights into immunogenomic dynamics, showing potential for combination immune checkpoint blockade in specific patient subgroups (75). Despite their potential, ICIs face challenges in TNBC, including low response rates in unselected patient populations and the development of resistance. Ongoing research aims to identify biomarkers such as PD-L1 expression to better predict response to ICIs.

Interestingly, the combination of ICIs with chemotherapy or radiotherapy can make the TME more conducive to immune activation, improving the clinical outcomes (76). For instance, the IMpassion130 study has demonstrated the efficacy of atezolizumab in combination with nab-paclitaxel, showing improved progression-free survival (PFS) in PD-L1-positive TNBC patients (77). Another multicenter phase II trial investigated the efficacy and safety of combining camrelizumab; an anti-PD-1 antibody, apatinib; a VEGFR2 tyrosine kinase inhibitor, and eribulin; a chemotherapy in patients with heavily pretreated advanced TNBC. This trial revealed that the combination therapy demonstrated promising efficacy with a manageable safety profile in this group of patients (78). Most recently, the TBCRC 043, a phase II randomized clinical trial, evaluated the efficacy of combining atezolizumab with carboplatin chemotherapy in patients with metastatic TNBC. Interestingly, the benefits of adding atezolizumab were observed regardless of PD-L1 status. Patients with high tumor-infiltrating lymphocytes (TILs), higher mutation burden, obesity, and uncontrolled blood glucose levels experienced greater benefits from the combination therapy. Notably, all TNBC subtypes benefited from the addition of atezolizumab, except for the luminal androgen receptor subtype (79). These trials have demonstrated improved progression-free survival, overall survival, and objective response rates, especially in patients with PD-L1-positive tumors, while also highlighting the potential for durable responses and manageable safety profiles, making this approach a promising treatment option in both early-stage and metastatic TNBC settings.

The Food and Drug Administration (FDA) of the United States has approved the following PD-1/PD-L1 inhibitors for the treatment of TNBC: pembrolizumab for high-risk early-stage triple-negative breast cancer, pembrolizumab for locally recurrent, unresectable, or metastatic TNBC, and atezolizumab for PD-L1-positive positive unresectable, locally advanced, or metastatic TNBC (80). Overall, ICIs represent a promising advancement in the treatment of TNBC, offering hope for improved outcomes in this difficult-to-treat cancer subtype. However, further studies are needed to optimize their use and broaden their benefits to more patients.

### 3.2 TAMs-targeting agents

Another immunological approach to target TNBC focuses on targeting tumor-associated macrophages (TAMs), in order to reprogram their tumor-promoting (M2-like) phenotype into a tumor-suppressing (M1-like) state or deplete TAMs to reduce their protumorigenic effects (81–83). It was stated that high densities of TAMs, particularly those expressing the CD163 marker, are associated with decreased overall survival and progression-free survival in breast cancer patients, with the prognostic impact varying by breast cancer subtype (84). In TNBC, the role of TAMs in tumor progression has been widely investigated, suggesting a potential benefit of targeting them. For example, a study showed that the combination of a chemotherapy agent, cyclophosphamide, with macrophage inhibition using either PLX3397 (a CSF1R inhibitor) or an anti-CSF1R antibody, led to increased infiltration of T and B cells into the tumor microenvironment and resulted in durable tumor regression in TNBC models (85). Moreover, a study revealed the combination of eganelisib, an oral immunomodulatory PI3K-γ inhibitor, with ICIs and chemotherapy reprogrammed tumor-associated macrophages to a cancer-fighting state, enhanced immune system activation, and remodeled the tumor environment to make it less supportive of cancer growth in TNBC. The results of this study are based on translational data derived from the MARIO-3 clinical trial (86). Another approach is using the chemokine receptors CCR2/CCR5 antagonists, which mediate the recruitment of monocytes to tumors where they differentiate into TAMs. For instance, a study suggested that inhibiting CCR2 can effectively reduce MCP-1-driven invasiveness and metastasis in TNBC by blocking the recruitment of pro-tumoral monocytes and macrophages to the tumor microenvironment (87). Further, inhibiting CCR5 through a novel antagonist targeting the CCL5/CCR5 axis suppresses tumor growth and metastasis in TNBC by regulating the CCR5-YAP1 signaling pathway, thereby disrupting pro-tumoral mechanisms (88). On the other hand, CD47-SIRPα pathway blockers were found to enhance the ability of macrophages to attack tumor cells, as it is stated that CD47 is overexpressed on cancer cells and inhibits macrophage-mediated phagocytosis (89). TNBC cells often express high levels of CD47, making this an attractive target. For example, PPAB001, a bispecific antibody targeting CD47 and CD24, enhances anti-PD-L1 efficacy in TNBC by reprogramming tumor-associated macrophages to an anti-tumoral M1 phenotype (90). Further, dual-targeting fusion protein against PD-L1 and CD47 inhibits TNBC by enhancing anti-tumor immunity and disrupting immune evasion mechanisms (91). Other approaches, like TLR agonists, can repolarize TAMs from an M2-like to an M1-like phenotype. An example of TLR agonists is Imiquimod (R837), which acts as a key immune activator in the nanoformulation, driving M1 macrophage polarization and synergizing with photothermal therapy to enhance the therapeutic efficacy against TNBC (92). While, bisphosphonates, such as zoledronic acid, were stated to effectively target TAMs in TNBC, reducing their pro-tumoral activity and inhibiting tumor growth and metastasis. Overall, targeting TAMs in TNBC is a promising strategy to reprogram the tumor microenvironment, reduce immune suppression, and enhance the efficacy of existing therapies against this aggressive cancer subtype. However, the effectiveness of TAM inhibitors as standalone treatments is limited, while combination therapies (e.g., chemotherapy, immunotherapy, or radiation) show greater potential. Further biomarker development is critical to identify patients who will benefit most from TAM-targeting therapies.

### 3.3 Anti-angiogenic agents

Angiogenesis is considered one of the main hallmarks of cancer, including TNBC (93). In tumors, the blood vessels are unevenly spread and disorganized. As a result, poor blood flow limits the delivery of chemotherapy and immunotherapy drugs to the inside of the tumor. It also prevents the removal of immune-suppressing cells from the TME (94). However, vascular endothelial cells lining the tumor express proteins like PD-L1, which activate regulatory T-cells and block the activity of cytotoxic T cells. Thus, the immunosuppressive environment of the tumor will be supported (95). In TNBC, the promotion of blood vessel growth in tumors is regulated by several factors, including VEGF, angiopoietin-2, placental growth factor, and TGF-β. All were reported to contribute to the tumor’s immunosuppressive environment in TNBC (96).

The anti-angiogenic agents targeting different angiogenic factors aim to inhibit tumor angiogenesis, normalize abnormal blood vessels, and improve the delivery of chemotherapy in the hypoxic TNBC microenvironment. These strategies aim to improve the immune system’s ability to fight TNBC (97). VEGF is the main driver of new blood vessel formation in cancer (98). Treatments that block VEGF pathways include monoclonal antibodies against VEGF, like bevacizumab (160), small-molecule tyrosine kinase inhibitors such as sorafenib (161), VEGF traps or decoy receptors like aflibercept (162), and VEGFR2 inhibitors such as ramucirumab (163, 164).

Anti-angiogenic therapy, primarily targeting VEGF signaling, represents a cornerstone approach for impairing tumor vascularization and inhibiting tumor growth (165). However, paradoxically, the blockade of VEGF pathways and subsequent disruption of tumor-associated vasculature often induce a hypoxic microenvironment within the tumor tissue in TNBC (56, 166). Upon inhibition of VEGF-mediated angiogenesis, tumors often experience a state of hypoxia due to the impaired formation of new blood vessels and the consequent reduction in oxygen supply. Hypoxia acts as a potent stressor in the TME, leading to the stabilization and activation of hypoxia-inducible factor-1α (HIF-1α), a key transcription factor that orchestrates cellular responses to low oxygen conditions (167). Activated HIF-1α induces the transcription of a variety of genes associated with survival, metabolism, angiogenesis, and immune modulation, including a range of pro-inflammatory and pro-angiogenic cytokines such as VEGF, interleukin-8 (IL-8), stromal cell-derived factor-1 (CXCL12), and transforming growth factor-β (TGF-β) (168–171).

The resultant cytokine surge within the TME facilitates tumor adaptation through several mechanisms (172). These cytokines activate alternative angiogenic pathways independent of VEGF, allowing the tumor to circumvent the blockade and restore a blood supply essential for its survival and proliferation (173). Furthermore, elevated cytokine levels modulate the immune microenvironment, fostering a more immunosuppressive and inflammatory milieu that favors tumor progression (174). Following anti-angiogenic therapy, these cytokine-driven changes also critically contribute to the development of drug resistance (94, 175, 176). Specifically, signaling pathways downstream of cytokine receptors, such as STAT3 (177), NF-κB (178), and PI3K/AKT (179), become persistently activated.

Collectively, these processes render tumors progressively less sensitive to both chemotherapeutic agents and continued anti-angiogenic therapy, ultimately culminating in multidrug resistance. Thus, while anti-angiogenic therapies initially restrict tumor growth, the induced hypoxic and inflammatory responses mediated via cytokines significantly undermine their long-term efficacy and highlight the need for combinatorial strategies to overcome resistance, the induced hypoxic and inflammatory responses mediated via cytokines significantly undermine their long-term efficacy and highlight the need for combinatorial strategies to overcome resistance (180, 181).

### 3.4 Other approaches to target TNBC’s TME

Since the cholesterol biosynthesis pathway is associated with TME responses and activities, there is a need to develop innovative inhibitors with low toxicity to target this metabolic pathway. Such novel inhibitors would offer a fresh therapeutic approach for treating the tumor microenvironment. The autophagy initiator LYN-1604 and myeloid cell leukemia-1 inhibitor S63845 are new targeted small-molecule medications that have been developed to cause cancer cell death (182, 183). Galunisertib, a TGF-β inhibitor, is now being studied in a clinical trial to determine the adverse effects and best dosage for treating patients with metastatic androgen receptor-negative TNBC (184). Moreover, tumor-infiltrating lymphocytes (TILs) are the major element in tumor cell immune infiltration in TNBC. TILs engage in interactions with tumor cells, alter the tumor immune microenvironment, and play a role in either immune suppression or attack by T1-cells. Therefore, therapeutic strategies like chimeric antigen receptor T (CAR-T) that encourage immune cell activation and penetration into tumor tissue show great potential. Another promising drugs for TNBC is antibody–drug conjugates (ADCs). The ability to target the treatment to tumor tissue rather than normal tissue is made possible by the identification between tumor cell antigens and antibodies. Due to certain characteristics of the extracellular or intracellular microenvironment (such as a low pH in the high metabolic TME), the entire ADC molecule is destroyed after recognizing the targeted antigens. ADCs provide an innovative, individualized therapeutic method with highly selective drug delivery. For patients with relapsed, refractory metastatic TNBC who have received at least two prior treatments, sacituzumab govitecan was the only ADC approved by the FDA in 2020 (68).

Despite the growing recognition of inflammation and cytokine signaling as central drivers of TNBC progression and therapy resistance, a deeper mechanistic understanding remains elusive. The dual roles of key cytokines such as TNF-α, TGF-β, IL-6, and IL-10, which act as both tumor suppressors and promoters depending on the tumor context, highlight the complexity of targeting these pathways therapeutically. While current strategies show promise, especially in preclinical settings, clinical translation remains limited by tumor heterogeneity, dynamic immune evasion, and the lack of predictive biomarkers for therapy responsiveness. Moreover, combinatorial therapies, such as immune checkpoint inhibitors with anti-angiogenic agents or TAM modulators, have yet to overcome resistance in a durable and broadly applicable manner. Future studies must move beyond observational correlations and aim to dissect temporal and spatial cytokine dynamics within the TME. Embracing integrative multi-omics and spatial transcriptomics approaches could illuminate the contextual behavior of cytokines and enable precision-targeted interventions. Ultimately, TNBC subtypes should be defined and stratified based not only on tumor-intrinsic features but also on immunologic and stromal landscapes. This will be crucial to overcoming current therapeutic limitations and improving patient outcomes.

While several TME-targeted therapies are in development, most focus on inhibiting single pathways such as PD-1/PD-L1 or STAT3. However, our proposed model suggests that TNBC resistance emerges from multi-node cytokine circuits, where immune, stromal, and tumor components reinforce each other through redundant signaling. This implies that combination strategies targeting both upstream cytokine receptors (e.g., IL-6R, TGFβR) and downstream effectors (e.g., STAT3, SMAD3, NF-κB) may be necessary to dismantle these feedback networks and reverse resistance.

### 3.5 Experimental models to study cytokine-driven drug resistance in TNBC

To better understand cytokine-mediated drug resistance and tumor progression, biologically relevant preclinical models are essential. Current therapies are limited by drug resistance and toxicity, underscoring the need for models that accurately reflect the TME and its complex cytokine networks (185). Preclinical models include traditional 2D cultures and advanced *in vitro* systems such as 3D spheroids, organoids, and co-cultures alongside *in vivo* models like xenografts, genetically engineered mice, and humanized systems enables more faithful modeling of cancer biology and supports the development of effective immunomodulatory therapies (186, 187).

In the context of TNBC, cytokine-mediated signaling plays a central role in shaping the TME and influencing therapeutic resistance (188). To dissect these pathways, a variety of experimental models have been developed, each offering unique advantages and limitations in modeling cytokine-driven processes. Conventional monolayer cultures of TNBC cell lines such as MDA-MB-231, BT-549 and Hs578T have been extensively used to study the effect of cytokine expression patterns and their signaling cascades on cancer hallmarks including proliferation, migration, and chemoresistance (189–192). However, these 2D models oversimplify the tumor environment, often lacking immune and stromal components crucial for cytokine crosstalk (193). To address this, researchers have turned to 3D spheroid cultures, tumor organoids, and co-culture systems that incorporate fibroblasts, macrophages, or lymphocytes (194, 195). These platforms better mimic the spatial organization and paracrine signaling of the native TME, enabling more accurate investigation of pro-inflammatory cytokines such as IL-6, IL-8, TNF-α, and TGF-β in drug resistance.

Furthermore, *in vivo* models offer essential insights into how cytokines modulate tumor behavior within a systemic physiological context. Orthotopic xenograft models using TNBC cell lines implanted into the mammary fat pad of immunodeficient mice allow for the study of cytokine-driven metastasis and treatment response *in situ* (196–198). More advanced models, such as humanized mouse models, enable the evaluation of immune–cytokine interactions in the presence of a human immune system, which is critical for testing immunotherapeutic strategies (199, 200). Similarly, patient-derived xenografts retain the cytokine expression profiles and heterogeneity of the original tumors, providing a powerful tool for translational studies (201, 202). Furthermore, innovative systems, such as zebrafish xenografts, have also emerged as dynamic models to study cytokine-mediated immune evasion and metastasis due to their optical transparency and compatibility with real-time imaging (203, 204).

These models, when combined with single-cell RNA sequencing and spatial profiling technologies, further enhance our ability to map cytokine networks and their functional impact within the evolving TNBC landscape. Overall, integrating multiple preclinical models is crucial to fully capture the complexity of cytokine-driven mechanisms in TNBC. This systems-based approach not only provides mechanistic insights but also facilitates the identification of novel targets for overcoming drug resistance and improving clinical outcomes.

## 4 Conclusions

The dynamic balance between pro-inflammatory and anti-inflammatory cytokines in TNBC plays a critical role in determining tumor progression. Dysregulation of cytokine signaling disrupts this balance, leading to an immunosuppressive TME that promotes immune evasion, tumor growth, and resistance to therapies. Cytokines such as TNF-α, TGF-β, IL-6, and IL-10 are pivotal players in these processes, driving inflammation, tumor progression, and immune suppression. Understanding the intricate interplay of cytokines within the TME is essential for developing targeted therapeutics that can combat immune suppression and enhance anti-tumor immune responses. Promising therapeutic strategies include inhibiting pro-inflammatory cytokines, encouraging the release of anti-tumorigenic cytokines, modulating the TME with cytokine inhibitors, and employing immune checkpoint inhibitors. These approaches hold significant potential to transform TNBC treatment by reducing inflammation, enhancing immune responses, and overcoming therapeutic resistance. However, further research is required to refine these strategies, unravel the complexities of the cytokine network, and develop personalized therapies. Harnessing the potential of cytokines as therapeutic targets may pave the way for novel treatment strategies, ultimately improving clinical outcomes and offering new hope for patients battling this aggressive breast cancer subtype. Future research should prioritize identifying key cytokine interaction hubs within the TME and developing multi-targeted interventions informed by such integrative mechanistic frameworks.
